# Comparative assessment of commercially available wound gels in ex vivo human skin reveals major differences in immune response-modulatory effects

**DOI:** 10.1038/s41598-022-20997-9

**Published:** 2022-10-19

**Authors:** S. Seiser, D. Cerbu, A. Gallhofer, J. Matiasek, A. Elbe-Bürger

**Affiliations:** 1 Department of Dermatology, Vienna General Hospital, Medical University of Vienna, Vienna, Austria; 2 Department of Plastic, Aesthetic and Reconstructive Surgery, St. Josef Hospital, Vienna, Vienna, Austria

**Keywords:** Medical research, Immunology, Cytokines, Infection, Inflammation

## Abstract

Wound healing is a crucial process for maintaining the function of human skin as a protective barrier to pathogens and other external stress factors. Hydrogels—in combination with antimicrobials—are often used, as moist wound care has been widely accepted as standard therapy. Recently, we reported about immune response-modulatory effects of an octenidine-based hydrogel, however little is known about the mechanism of action of other hydrogels including antiseptic molecules or chlorine-based and chlorine-releasing agents, respectively. The aim of this study was the comparative assessment of commercially available wound gels (octenilin^®^, Prontosan^®^, Lavanid^®^, Betadona^®^, ActiMaris^®^, Microdacyn_60_^®^, Veriforte^TM^med) with regard to their effects on the secretion of distinct cytokines (IL-6, IL-8, IL-10), matrix-metalloproteinases as well as their potential to cause alterations in skin structure and apoptosis. Hence, tape-stripped human ex vivo skin biopsies were treated topically with wound gels and cultured for 48 h. Enzyme-linked immunosorbent assays and an enzyme activity assay of culture supernatants revealed that octenilin^®^ demonstrates significantly broader anti-inflammatory and protease-inhibitory capacities than other wound gels. Further, haematoxylin & eosin as well as caspase-3 staining of treated biopsies showed that octenilin^®^ does not alter skin morphology and shows the least interfering effect on human epidermal cells compared to untreated controls. Overall, this study clearly demonstrates totally different effects for several commercially available hydrogels in our wound model, which gives also new insight into their tissue compatibility and mode of action.

## Introduction

As one of the body’s largest organs human skin is not merely a structural barrier protecting organisms from a diversity of environmental insults but has a variety of other crucial functions. Beside temperature control, water content regulation, vitamin D production and energy storage, it harbors one of the most sophisticated sensory networks in the body and is an active immune organ in both antimicrobial immunity and homeostatic processes like wound healing^1–3^. Being constantly exposed to potential injury, the ability of human skin to heal and restore its barrier function is a tremendously important process for the survival of all higher organisms^4^. Cutaneous wound healing in adult mammals is a complex, multi-step process which can be divided into four overlapping phases: (i) haemostasis and coagulation, (ii) inflammation, (iii) proliferation and repair, as well as (iv) remodelling and maturation, during which an excessive amount of communication between all cellular components is essential^5,6^. The cytokines IL-6 and IL-8 play a major role in the first phases of physiological wound healing, while an increased expression can lead to a shift towards an inflammatory state, in which chronic wounds are often trapped^7^. In fetal skin, which is known to heal with almost no signs of inflammation, diminished expression levels of IL-6 and IL-8 were shown compared to adult skin^8^. During the remodelling phase matrix-metalloproteinases (MMPs) have a major role as they are dissolving the basal lamina and lysing surrounding tissue, so that proliferation and migration can take place. The major regulators of MMPs are the tissue inhibitors of metalloproteinases (TIMPs)^9^. Disturbance in the balance between MMPs and TIMPs can lead to impaired wound healing, inflammation and even scar formation^10,11^. Aiming to avoid infections or compromised wound healing, the development of different wound dressings is still in the focus of research. Hydrogels can improve wound healing as they have a beneficial impact on the final outcome and are currently state of the art. Accordingly, a wide variety of products are available. These include “plain” hydrogels which can also be used as a conveyance vehicle for several bioactive compounds^12^. Among them are mainly antiseptic molecules like octenidine, polyhexanide or povidone-iodine, but also chlorine-based and chlorine-releasing agents, respectively^13,14^. Although these hydrogels are frequently used nowadays in modern wound management, a detailed evaluation of their mechanism of action is still lacking. Octenidine is a well-established antimicrobial molecule for human skin, mucous membrane, and wound antisepsis showing antibacterial, antifungal and partially antiviral properties^15,16^. Octenilin^®^ wound gel (Oct) is commonly used for the treatment of chronic wounds, having the ability to clear bacterial biofilms^17^. Further, studies revealed that Oct has beneficial effects on scar quality when applied as post-operative wound dressing^18^. Ex vivo studies with human skin demonstrated that Oct (i) prevents emigration of Langerhans cells which are crucial cells for protection and homeostasis of the epidermis, (ii) decreases the secretion of distinct cytokines (IL-6, IL-8, IL-10), and (iii) inhibits the secretion of particular tissue MMPs^19,20^. To test whether these properties are specific for Oct or are also valid for other wound gels, an established ex vivo human wound model^19–21^ combined with subsequent local treatment of wounded skin with selected wound gels were applied (Table 1). Evaluation included histological and immunofluorescence staining of treated biopsies as well as analysis of a selected panel of cytokines and MMP activity in respective culture supernatants.

**Table 1:** Tested commercially available wound gels.

Wound gels	Composition	Manufacturer	Classification
Octenilin^®^ (Oct)	0.05% octenidine, water, propylene glycol, hydroxyethyl cellulose	Schülke & Mayr GmbH, Norderstedt, DE	Medical device class IIb
Prontosan^®^ (Pro)	0.1% polyhexanide, 0.1% betaine surfactant, glycerol, water, hydroxyethyl cellulose	B. Braun Medical AG, Sempach, CH	Medical device class IIb
Lavanid^®^ (Lav)	0.04% polyhexanide; Ringer's solution, glycerin, macrogol, hydroxyethyl cellulose	Serag-Wiessner GmbH & Co. KG, Naila, DE	Medical device class IIb
Betadona^®^ (Bet)	1% PVP-iodine; macrogol, sodium hydrogen carbonate, water	Mundipharma GmbH, Wien, AT	Medicinal product
ActiMaris^®^ (Act)	3% sea salt (Sal Maris), 0,2% sodium hypochlorite, water, lithium-magnesium-sodium-silicate, sodium oxychloride	ActiMaris AG, Appenzell, CH	Medical device class IIb
Microdacyn_60_^®^ (Mic)	Water, sodium chloride, sodium magnesium fluorosilicate, sodium phosphate, sodium hypochlorite, hypochlorous acid	Oculus Technologies of Mexico S.A. de C.V., Guadalajara, MX	Medical device class IIb
Veriforte™med (Ver)	Water, sodium chloride, hypochlorous acid, sodium hypochlorite, colloidal silicate	P.G.F. Industry Solutions GmbH, Elixhausen, AT	Medical device class IIb

## Methods

### Ethics statement

Skin was obtained from twelve healthy female patients (age range of panel 1: 20–45 years; age range of panel 2: 30–47 years) that have undergone routine plastic surgery procedures. Abdominal skin has been chosen, as it is the region most frequently obtained during plastic surgery in the General Hospital of Vienna, and most importantly, provides sufficient tissue for the required number of biopsies. In addition, we focused on female Caucasian donors aged between 20 and 47 years to minimize the effect of gender, ethnicity and age in our study. Informed consent was obtained from all donors and a valid ethics vote is available, approved by the ethics committee of the Medical University of Vienna (EK Nr. 1969/2021). Further, we confirm that all experiments were performed in accordance with relevant guidelines and regulations.

### Superficial ex vivo human skin wound model

Freshly isolated skin (1–2 h after surgery) was disinfected using Kodan Forte (Schülke & Mayr GmbH, Norderstedt, DE). D101-Squame standard self-adhesive discs (CuDerm, Dallas, US) were applied onto excised skin with constant pressure for 10 s and removed from top down. This procedure was executed on the same location 50 times in a row. Subsequently, the tape-stripped (TS) skin was dermatomed (0.6 mm; Acculan^®^ 3Ti; Aesculap Inc., Center Valley, US) and biopsies (8 mm; Kai Europe GmbH, Solingen, DE) were taken.

### Topical application of wound gels and culture conditions

Before culture, commercially available wound gels (Table 1), were applied topically onto TS skin biopsies (50 µl/biopsy) or left untreated and cultured in 12-well culture plates (TPP, Trasadingen, CH) in the presence of DMEM (Gibco, Carlsbad, US), supplemented with 10% fetal bovine serum (FBS, Gibco, Carlsbad, US) and 1% penicillin–streptomycin (Gibco, Carlsbad, US) at 37 °C and 5% CO_2_ (Heracell™ 150i CO_2_ Incubator; Thermofisher Scientific, Waltham, US). After 48 h of incubation, culture supernatants were collected and stored at − 80 °C for enzyme-linked immunosorbent assays (ELISA) as well as an enzyme (MMP) activity assay, and the biopsies were fixed in 7.5% formaldehyde (SAV Liquid Production, Flintsbach a. Inn, DE) for further analysis.

### ELISA

Culture plates (Nunc Immuno 96 well flat-bottom culture plate; MercK KGaA) were coated with capture human antibodies IL-8 (Thermo Fisher Scientific), IL-6, IL-10 and MMP2 (R&D Systems) overnight at 4 °C or room temperature. On the next day, plates were washed with washing buffer (0.05% Tween in PBS) and blocked with blocking buffer (4% BSA, 0.5% Tween in PBS) or reagent diluent (1% BSA in PBS). Another washing step was performed and standards and samples were pipetted on the plates in an appropriate dilution and incubated for 2 h. Next, plates were aspirated and detection antibody was applied (Thermo Fisher Scientific #M802B, R&D Systems,) and incubated for another 2 h. Then, plates were washed again and streptavidin-horse-radish-25 peroxidase (Thermo Fisher Scientific #34028, R&D Systems) was applied. After an incubation time of 20–30 min, another washing step was performed and 3,3′,5,5′-tetramethylbenzidine substrate reagent A + B (BD Biosciences, USA) was applied and incubated for 20 min. Subsequently, a stop solution (2 N H_2_SO_4_) was added and measurement was performed immediately at 450 nm with a photometer (MultiskanTM FC Microplate Photometer; Thermofisher, USA).

### Histochemistry

Paraffin sections (5 µm) on Superfrost™ slides (Thermofisher Scientific, Waltham, US) were stained with hematoxylin and eosin (H&E) according to standardized protocols at the General Hospital Vienna, Department of Pathology.

### Enzyme activity assay

The activity of MMPs in culture supernatants was measured using the AmpliteTM Universal Flourometric protease activity Assay Kit (AAT Bioquest, USA). Proteases in supernatants were activated with 4-aminophenylmercuris acetate (2 mM) for 3 and 24 h. Activated supernatants and controls were pipetted in duplicates into a flat-bottom culture plate (100 μl/well; Coning^®^ 96 well White Polystyrene Microplate; Merck KGaA) and MMP GreenTM substrate (50 μl/well; AAT Bioquest) was added. Fluorescence intensity was monitored as kinetic measurement with a plate reader (BMG FLUOstar OPTIMA Microplate Reader; BMG Labtech Inc. USA) at Ex/Em = 490/525 nm.

### Caspase-3 staining and quantification

To test apoptosis in skin cells, tissue sections on immunohistochemistry (IHC) FLEX microscope slides (Dako Denmark, Glosturp, DK) were incubated with a cleaved caspase-3 antibody (#MAB835; RD Systems Donji, Kneginex, HR) at 4 °C overnight in a wet chamber. The next day, the primary antibody was removed, slides were washed in PBS and incubated with Alexa Fluor™ 546 goat anti-rabbit antibody (#PA5-16891; Thermofisher Scientific, Waltham, US) at room temperature for 1 h. After incubation, the secondary antibody was removed, the slides were washed in PBS and incubated with 4′,6-diamidino-2-phenylindole (DAPI) for 1 min. After another washing step, slides were mounted (Fluoromount-G, SouthernBiotech, Birmingham, US) covered (Menzel-Gläser Coverslips, Thermofisher Scientific, Waltham, US) and stored at 4 °C. To quantify caspase-3^+^ cells, 4 fields of view (FOV) per slide have been counted using an Olympus AX70 microscope (Olympus, Tokyo, JP). Pictures were obtained via Spot RT3 camera (Visitron Systems GmbH, Puchheim, DE) and MetaMorph software (Molecular Devices, San Jose, US).

### Statistical analysis

ELISA and activity assay data was statistically analysed using GraphPad Prism 9.3.1. A Wilcoxon matched-pairs signed rank test was performed (n = 6). Data is presented as a mean ± standard deviation (SD) and results were considered statistically significant with p-values smaller than 0.05. Counted numbers of caspase-3^+^ cells were statistically analysed using GraphPad Prism 9.3.1. As each FOV can be seen as a technical replicate, the mean of all 4 FOVs was determined, and a two-way analysis of variance (ANOVA) was performed. Data is presented as a mean ± SD. Results were considered statistically significant with p-values smaller than 0.05.

## Results

### Oct has a significantly broader and higher inhibitory capacity for a panel of cytokines and proteases on wounded human skin compared to other wound gels

**Figure 1:**
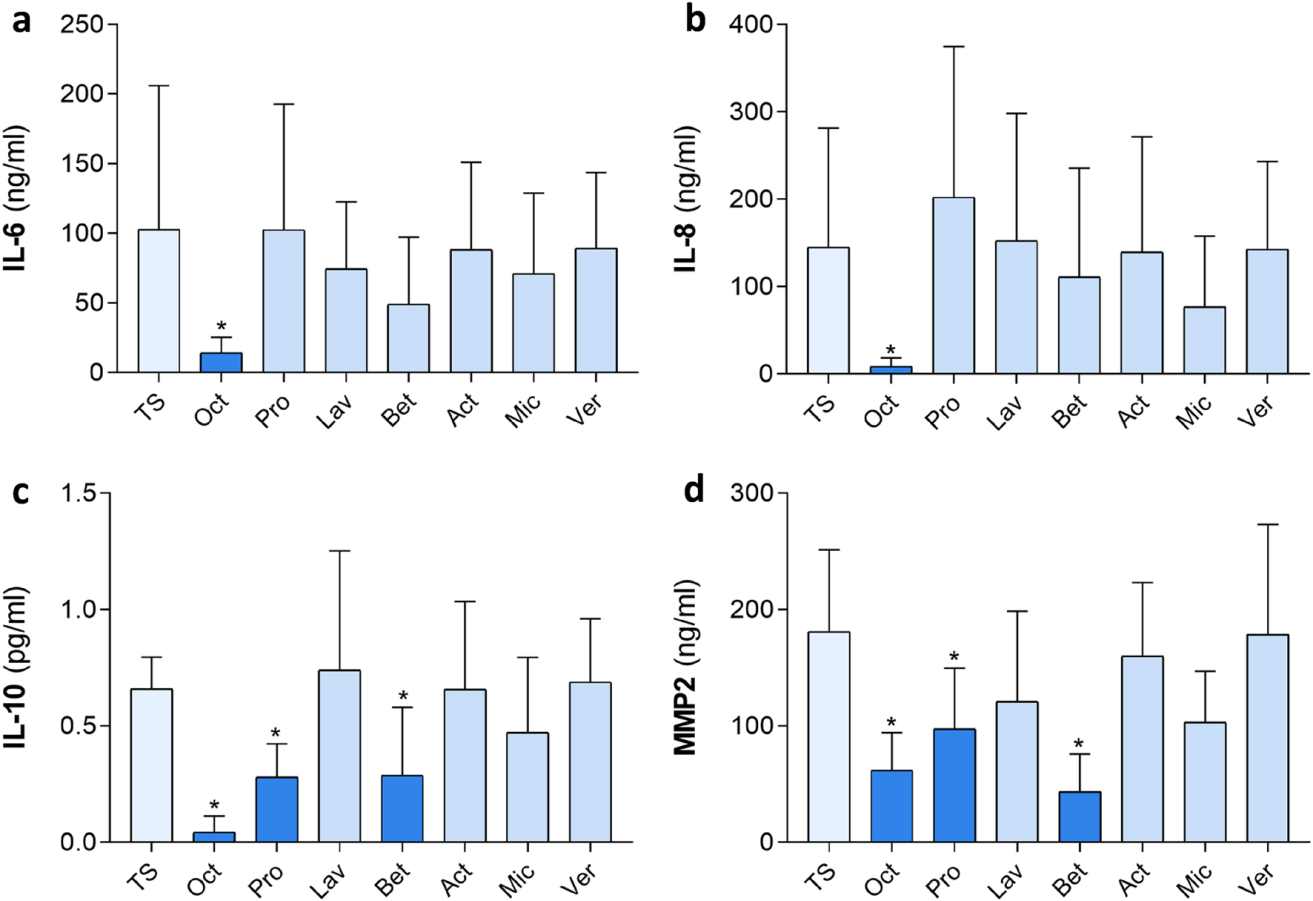
Quantification of cytokines in superficially wounded ex vivo skin treated with commercially available wound gels. Shown are (**a**) IL-6, (**b**) IL-8, (**c**) IL-10, (**d**) MMP2 concentration levels in culture supernatants of 48 h cultured human tape-stripped (TS) skin biopsies topically treated with indicated wound gels as well as a TS untreated control. Samples were analysed in duplicates with an enzyme-linked immunosorbent assay (ELISA). Data is presented as a mean ± standard deviation. A Wilcoxon matched-pairs signed rank test was performed with GraphPad Prism 9.3.1. *p ≤ 0.05; n = 6, female donors, location: abdomen, age range: 20–45 years (panel 1).

We reported recently that octenilin^®^ wound gel (Oct) significantly inhibits the secretion of particular cytokines and MMPs in a superficial human skin wound model^19,20^. In this study, the effectiveness of Oct was compared with the efficacy of other antiseptic wound gels such as Prontosan^®^ (Pro), Lavanid^®^ (Lav), Betadona^®^ (Bet), ActiMaris^®^ (Act), Veriforte™med (Ver) and Microdacyn_60_^®^ (Mic) (Table 1). Therefore, each wound gel was applied topically onto TS abdominal skin biopsies of six healthy female donors (panel 1) and cultured for 48 h. Assessment of respective culture supernatants using ELISA revealed that IL-6 and IL-8 levels were significantly diminished only in culture supernatants of skin biopsies treated with Oct, while treatment with any other wound gel had no effect (Fig. 1a,b). IL-10 and MMP2 concentrations were significantly lower in culture supernatants of skin samples treated with Oct, Pro and Bet compared to untreated TS controls, Lav, Act, Mic and Ver (Fig. 1c,d).

**Figure 2:**
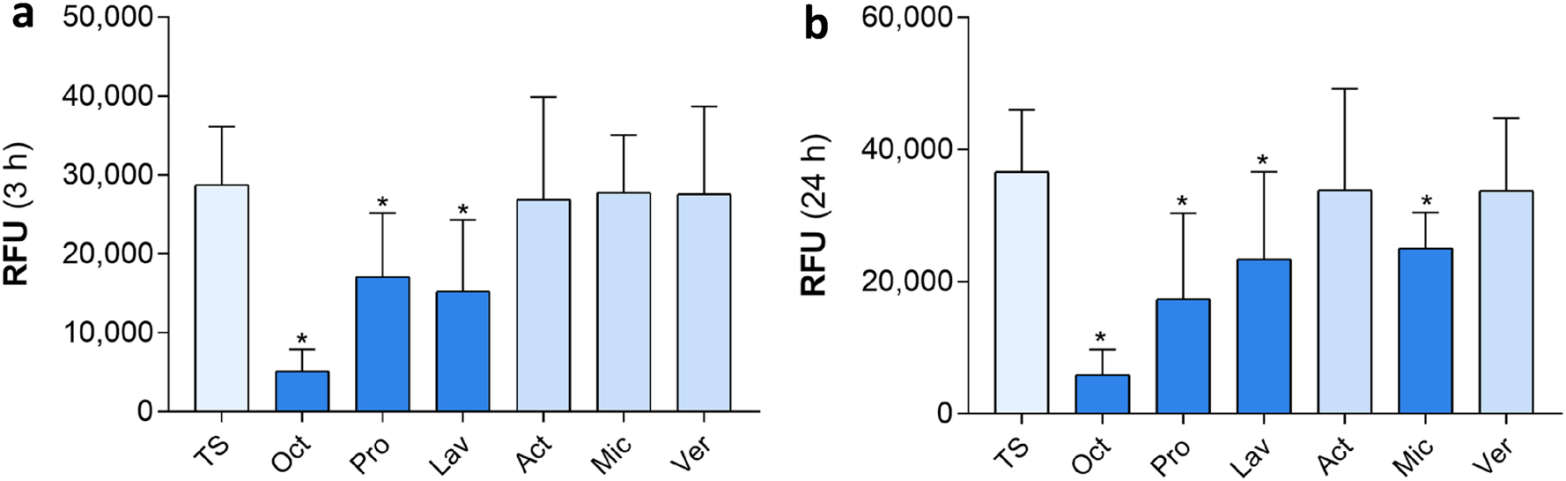
Not all wound gels inactivate MMPs in wounded human ex vivo skin. Shown are activated (**a**) MMP1, MMP2, MMP8, MMP9, MMP12, MMP14 (3 h activation), (**b**) MMP3, MMP10 (24 h activation) concentration levels in culture supernatants of 48 h cultured human tape-stripped (TS) skin biopsies treated with indicated wound gels as well as a TS untreated control. Samples were analysed in duplicates with an MMP activity assay. Relative fluorescence units (RFU) are indicated. Data is presented as a mean ± standard deviation. A Wilcoxon matched-pairs signed rank test was performed with GraphPad Prism 9.3.1. *p ≤ 0.05; n = 6, female donors, location: abdomen, age range: 20–45 years (panel 1).

MMPs and their inhibitors play a crucial role in tissue remodeling as they are capable of degrading extracellular matrix. However, an imbalance or prolonged expression of MMPs can lead to an impaired wound healing^11,22^. Oct has been shown to have an inhibitory effect on various tissue MMPs in wounded human ex vivo skin^20^. Using an MMP activity assay revealed that not only Oct, but also Pro and Lav significantly inhibited MMPs that are activated within three hours, including MMP1, MMP2, MMP8, MMP9, MMP12 and MMP14 (Fig. 2a). The same wound gels (Oct, Pro, Lav) and additionally Mic had a significantly inhibitory effect on MMPs that are activated within 24 h, including MMP3 and MMP10 (Fig. 2b). Of note, Bet had to be excluded from this experiment as povidone-iodine gives the gel a brownish colour, which unfortunately interferes with the assay.

### With exception of Oct and Lav all wound gels alter the epidermal architecture

**Figure 3:**
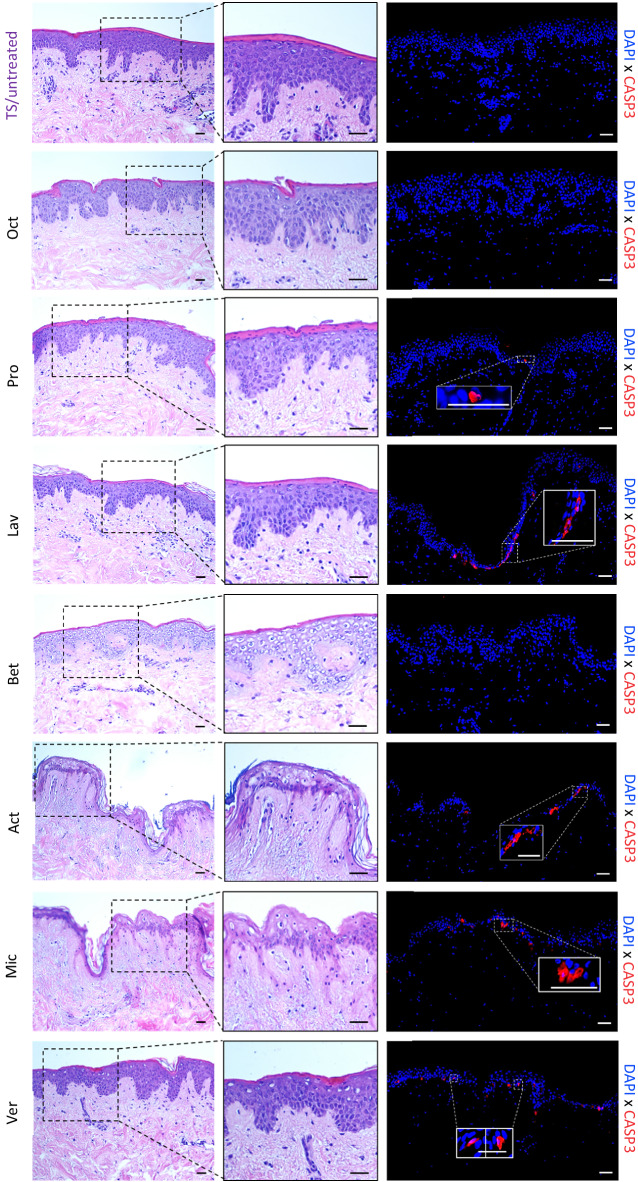
Assessment of the skin structure and apoptosis upon treatment with wound gels. Representative images and zoom-ins of the boxed areas of haematoxylin and eosin (H&E)-stained tape-stripped (TS) sections from biopsies (age 38 years) either left untreated or treated with indicated wound gels using a light microscope. The presence of caspase-3 expressing cells was evaluated using a caspase-3 (CASP3) antibody and visualisation with a secondary Alexa Fluor™ 546 AB (red). Nuclei were counterstained with DAPI (blue). Scale bar = 50 μm.

For histological examination of skin treated with different wound gels, abdominal skin from another panel of six healthy female donors (panel 2) within the same age range as panel 1 has been collected. Aiming to approve biological similarities, culture supernatants of treated skin biopsies from both panels were tested for IL-8 concentrations using ELISA. As the results of both panels revealed a robust concordance (Fig. S1), we next examined the morphology and skin structure of the treated biopsies via H&E staining. To assess whether treatment with any of the wound gels (Table 1) induced apoptosis, immunofluorescence (IF) staining of paraffin sections with an antibody directed against caspase-3, the principal apoptosis-associated effector caspase, was employed^23^. One representative donor for both staining procedures is shown (Fig. 3). Evaluation of H&E stained sections of biopsies of all six donors, uncovered abnormalities in skin structure depending on the applied wound gels. The most striking alteration in various treated biopsies was observed in the cytoplasm of epidermal cells, where vacuolisation occurred. Biopsies treated with Bet revealed a clear cut altered epidermal structure within all donors, showing excessive vacuolisation of the cytoplasm of almost all keratinocytes and most likely other epidermal cell types (e.g. Langerhans cells, T cells, melanocytes) in all *strata* (Fig. 3, Table 2). Skin sections of biopsies treated with Act or Mic regularly showed severe vacuolisation, often, but not exclusively, in apical layers (Fig. 3, Table 2). Of note, in biopsies treated with Act and Mic demarcation of the different epidermal layers was hardly possible in some areas (Fig. 3). Skin sections treated with Pro and Ver regularly showed moderate vacuolisation of keratinocytes, often in apical epidermal layers, being in close proximity to the remains of the *stratum corneum* upon TS and therefore the treatment side (Fig. 3, Table 2). Lav- and Oct-treated skin sections displayed highest resemblance with the TS untreated control (Fig. 3, Table 2), showing sporadic cytoplasmic cell vacuolisation. The abundance and severity of vacuolisation for each wound gel is summarized in Table 2.

**Table 2:** Skin biopsies treated with different wound gels and severity of induced vacuolisation.

Wound gels	Severity of vacuolisation^a^
Bet	High: donors 1–4
Mic	High (apical): donors 1, 3, 4; high (basal): donor 2
Act	High (apical): donors 1, 2, 3; moderate (apical): donor 4
Pro	Moderate (apical): donor 1; moderate–high (apical): donors 2, 3
Ver	Moderate–high (apical): donors 1, 3; moderate: donor 2
Lav	Mild (individual cells): donors 1, 2
Oct	Mild (individual cells): donors 1, 3

^a^Most severe to least severe (mild).

### Oct and Bet do not induce apoptosis in comparison to other wound gels

**Figure 4:**
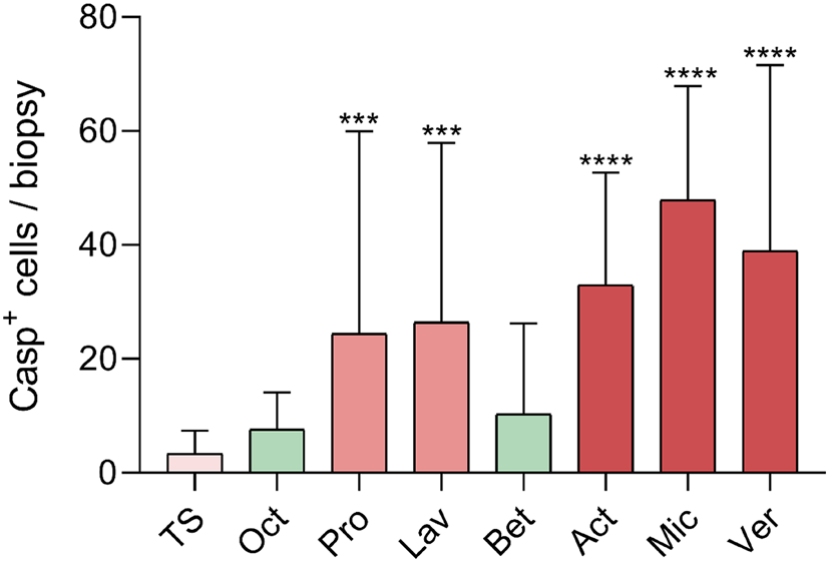
Numbers of caspase-3^+^ cells in wounded human ex vivo tissue upon wound gel treatment. Shown is the number of caspase-3^+^ cells in treated skin biopsies counted within 4 fields of view (FOV) per biopsy (tape-stripped (TS), untreated control and treated with Oct, Pro, Lav, Bet, Act, Mic or Ver) of six donors. FOVs are assessed as technical replicates and data is presented as a mean ± standard deviation. Two-way analysis of variance (ANOVA) was performed using GraphPad Prism 9.3.1. ***p ≤ 0.001, ****p ≤ 0.0001.

In situ staining and quantification revealed only occasionally present or no caspase-3^+^ cells in sections treated with Oct and Bet in all six donors and was comparable with the untreated TS control (Figs. 3 and 4). These data imply that Oct and Bet do not have an apoptotic effect when applied locally on cultured skin biopsies within the observation period of 48 h. In contrast, biopsies treated with Pro, Lav, Act, Mic and Ver show significantly more caspase-3^+^ cells per section than the TS control (Figs. 3 and 4).

## Discussion

In comparison to standard dry wound dressings, moist wound healing evoked by the use of hydrogels results in faster and better quality of healing, which can be further enhanced by addition of different bioactive compounds^12,15^. Apart from the broad antimicrobial effect of the antiseptic molecule octenidine, the hydrogel Oct has been proven to (i) promote the healing of (chronic) wounds in various clinical settings^17,24–27^, (ii) decrease the incidence of scar hypertrophy in vivo^18^, and (iii) act cytokine- as well as protease-inhibitory in wounded human and porcine ex vivo skin^19,20,28^. As a detailed evaluation of the mechanisms of other clinically used wound gels is still lacking, six commercially available wound gels were comparatively assessed with Oct using an established human ex vivo skin model. To obtain most accurate data, as many parameters as possible working with human tissues were standardised, such as choosing the same sex (female), equal body location (abdomen), defined age range (20–47 years), specified biopsy thickness (0.6 mm) and biopsy size (8 mm in diameter).

IL-6 is produced at sites of inflammation and plays an important role in acute phase response, enhancing the inflammatory response by inducing IL-8 concentrations at the wound site^29^. In contrast to adult wound healing, fetal wound healing is accompanied with minimal inflammation and virtually scarless repair. One of the potential reasons for this phenomenon is the fact that IL-6 and IL-8 are significantly decreased in fetal skin compared with postnatal skin^30,31^. Additionally, it has been shown that IL-8 levels are elevated in slowly healing burn wounds as well as in fibroblasts that derived from keloid scar, again proving its potential role in impaired wound healing and scar formation^32,33^. By studying these cytokines, we not only confirmed our previous findings^19,20^, showing that Oct significantly reduced IL-6 and IL-8 secretion when applied topically on wounded ex vivo human skin (Fig. 1), but also demonstrated for the first time the uniqueness of this beneficial effect, as none of the other tested wound gels showed this effect.

During wound healing the cytokine IL-10, known to have anti-inflammatory as well as pro-inflammatory properties^34^, represses the pro-inflammatory cascade and is even thought to play a major role in the transition from anti-inflammatory pre-natal wound healing to pro-inflammatory post-natal wound healing^35^. However, IL-10 is a pleomorphic cytokine with various phenotypic effects and multiple functions in organ fibrosis known to have also maladaptive effects^36^. It has been shown that the development of a T_H_2 response with production of IL-10 (amongst others) has been strongly linked to fibrogenesis^37^. Further, a study demonstrated the percentage of IL-4 and IL-10-producing T cell subsets was significantly elevated in the serum of burn patients compared to serum from healthy volunteers^38^. In our study, we found that besides Oct two other hydrogels, Pro and Bet, reduce IL-10 levels in TS human skin (Fig. 1). Our observation that Pro and Bet have an effect on IL-10 but not on IL-6 and IL-8 could be explained by the fact that the latter cytokines belong to a different cytokine family and thus show a different effector profile. As Oct, Pro and Bet contain different active compounds (Table 1) another mechanism of action is not surprising and remains to be further investigated.

MMPs are other potential candidates involved in cutaneous wound healing, capable of dissolving extracellular matrix, which is an important process during the remodeling phase. A dysregulation of MMP levels leads to excessive extracellular matrix degradation and alteration of the cytokine profile leading to delayed wound closure^39^. Further, it has been demonstrated that MMP2 mRNA expression is significantly increased in different types of human scar tissue^10^. We found decreased MMP2 levels in supernatants of skin biopsies treated with Oct, Pro and Bet, resembling the results for IL-10 (Fig. 1). Further examination of various tissue MMPs with an enzyme activity assay showed that Oct, Pro and Lav had an inhibitory effect on a variety of MMPs (MMP1, MMP2, MMP8, MMP9, MMP12, MMP14), mostly collagenases and gelatinases, that are activated within a time range of three hours. The same hydrogels, together with Mic inhibited MMP3 and MMP10, which are activated within 24 h and belong to the family of stromelysins (Fig. 2). Our findings suggest that among all wound gels, Oct is the only gel that has a significant effect on all tested proteins and therefore a broader anti-inflammatory as well as protease-inhibitory capacity than the other tested hydrogels (Table 3).

**Table 3:**
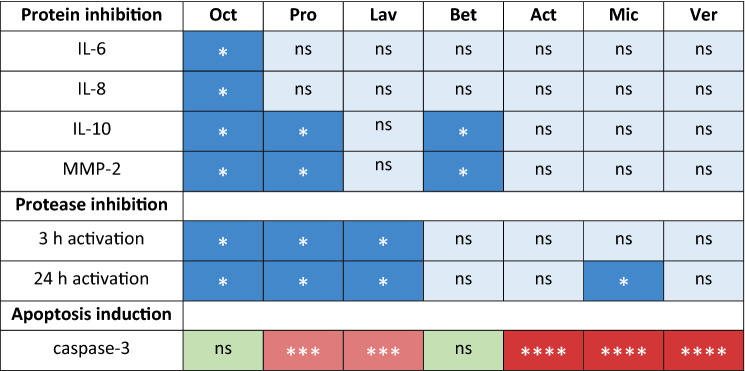
Overview—effects of all tested wound gels compared to untreated control.

A Wilcoxon matched-pairs signed rank test and a two-way analysis of variance (ANOVA) were performed for protein/protease inhibition data and for caspase-3 positive cell counts, respectively with GraphPad Prism 9.3.1. *p ≤ 0.05, ***p ≤ 0.001, ****p ≤ 0.0001, ns = not significant.

Analogous to our previous finding^19^ Oct did not alter the skin architecture and was comparable with the TS untreated control (Fig. 3). With exception of Lav, all other wound gels displayed alterations in epidermal cells, most notably vacuolisation of the cytoplasm (Fig. 3). Vacuolisation is a morphological phenomenon in which cells display an abnormal retention of water. This phenomenon often occurring after exposure to bacterial or viral pathogens, as well as to natural and artificial compounds, is a stress factor and frequently but not necessarily associated with cell death^40^. Overall, skin biopsies treated with Pro and Ver displayed moderate vacuolisation, but Act and Mic regularly showed severe vacuolisation (Fig. 3, Table 2). While the epidermis in biopsies with only mild vacuolisation appears similar to the one of TS untreated skin, demarcation of epidermal *strata* is often not possible in areas of severe vacuolisation. As vacuolisation often happened in apical epidermal *strata*, close to the treatment area, it is likely that components of the hydrogels attain underlying keratinocytes, which might be detrimental to a certain extent. Almost in line with our histological data are those obtained using the apoptosis indicator caspase-3. Oct and Bet did not show any caspase-3^+^ cells which was comparable to the untreated TS control, whereas the remaining wound gels (Pro, Lav, Act, Mic and Ver) displayed at least one caspase-3^+^ cell per section (Fig. 3) and was confirmed by enumerating caspase-3^+^ cells in skin sections. As no significant increase in the number of caspase-3^+^ cells was observed in Oct and Bet treated sections compared to untreated controls, our data imply that they do not have significant apoptotic effects during the observation period. Pro and Lav, which both contain polyhexanide as antiseptic molecule, significantly and similarly enhanced the number of caspase-3^+^ cells in treated skin sections. Act, Mic and Ver, which are all hydrogels based on hypochlorous acid, caused even higher numbers of caspase-3^+^ cells and are the hydrogels with the most remarkable apoptotic effects. Of note, a direct comparison between vacuolisation events and the number of caspase-3^+^ cells is not applicable, as we do not know if the mechanism behind cytoplasmic vacuolisation in this experiment indeed leads to cell death. In line with this assumption is our observation with Bet, indicating that caspase-3 induced apoptosis is presumably not involved in vacuolisation of epidermal cells, as biopsies treated with Bet show severe vacuolisation, while they reveal almost no caspase-3^+^ cells. Nevertheless, these vacuolisation events demonstrate a severe alteration of the skin cell architecture and might even cause an impairment of the physiological barrier function of human skin.

To conclude, the current study confirms and extends our knowledge about the efficacy and beneficial effects of Oct in comparison to other wound gels (Table 3). In addition to its unique pro-, as well as anti-inflammatory and protease-inhibitory effect, Oct does neither alter skin structure, nor cause caspase-3 induced apoptosis of epidermal cells, thus providing a safe option for wound dressings in clinical conditions.

## Supplementary Information

Supplementary Legends.

Supplementary Figure S1.

## Data Availability

Data, in anonymous format (according to data protection policy in the ethics agreement) is available on reasonable request.
