# Supplementary material for: Comparative assessment of commercially available wound gels in ex vivo human skin reveals major differences in immune response-modulatory effects
Source: Sci Rep. 2022 Oct 19;12:17481. doi: 10.1038/s41598-022-20997-9 (PMC9581930; doi:10.1038/s41598-022-20997-9)
Supplement: Supplementary file 1 — Supplementary Legends. [file 41598_2022_20997_MOESM1_ESM.docx]

**Figure S1: Comparative assessment of IL-8 secretion patterns of wound gel treated human skin biopsies derived from two different donor panels.**

Shown are IL-8 concentrations in culture supernatants of 48 hour cultured human tape-stripped (TS) skin biopsies treated with indicated wound gels as well as a TS untreated control. **a**) n = 6, female donors, location: abdomen, age range: 20-45 years (panel 1); **b**) n = 6, female donors, location: abdomen, age range: 30-47 years (panel 2). Samples were analysed in duplicates with an enzyme-linked immunosorbent assay (ELISA). Data is presented as a mean ± standard deviation. A Wilcoxon matched-pairs signed rank test was performed with GraphPad Prism 9.3.1. *p ≤ 0.05.
