# Supplementary figures and images for: Comparative assessment of commercially available wound gels in ex vivo human skin reveals major differences in immune response-modulatory effects
Source: Sci Rep. 2022 Oct 19;12:17481. doi: 10.1038/s41598-022-20997-9 (PMC9581930; doi:10.1038/s41598-022-20997-9)

**Supplementary Figure 1**


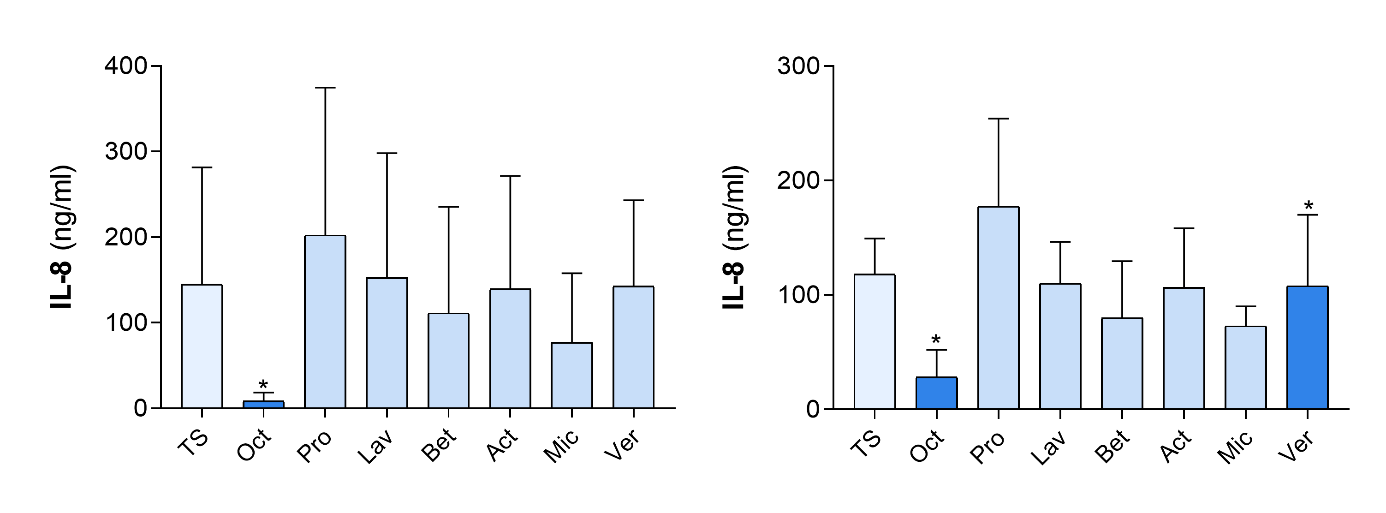


**b**

**a**

Supplement: Supplementary file 2 — Supplementary Figure S1. [file 41598_2022_20997_MOESM2_ESM.docx]
